# Short-term predictive ability of selected cardiovascular risk prediction models in a rural Bangladeshi population: a case-cohort study

**DOI:** 10.1186/s12872-016-0279-2

**Published:** 2016-05-26

**Authors:** Kaniz Fatema, Bayzidur Rahman, Nicholas Arnold Zwar, Abul Hasnat Milton, Liaquat Ali

**Affiliations:** Department of Epidemiology, Bangladesh University of Health Sciences (BUHS), 125/1, Darus Salam, Mirpur, Dhaka-1216 Bangladesh; The School of Public Health and Community Medicine, Faculty of Medicine, The University of New South Wales, Sydney, NSW 2052 Australia; Centre for Clinical Epidemiology and Biostatistics (CCEB), The School of Medicine and Public Health, Faculty of Health, The University of Newcastle, Newcastle, NSW 2008 Australia; Department of Biochemistry and Cell Biology, BUHS, 125/1 Darus Salam, Mirpur, Dhaka-1216 Bangladesh

**Keywords:** Cardiovascular disease, Coronary heart disease, Myocardial infarction, Framingham risk scores, Case-cohort, CVD risk prediction, Bangladesh, NB-NCDP

## Abstract

**Background:**

Prediction of absolute risk of cardiovascular diseases (CVDs) has important clinical and public health significance, but the predictive ability of the available tools has not yet been tested in the rural Bangladeshi population. The present study was undertaken to test the hypothesis that both laboratory-based (Framingham equation and WHO/ISH laboratory-based charts) and non-laboratory-based tools may be used to predict CVDs on a short-term basis.

**Methods:**

Data from a case-cohort study (52989 cohort and 439 sub-cohort participants), conducted on a rural Bangladeshi population, were analysed using modified Cox PH model with a maximum follow-up of 2.5 years. The outcome variable, coronary heart diseases (CHDs), was assessed in 2014 using electrocardiography, and it was used as a surrogate marker for CVDs in Bangladesh. The predictive power of the models was assessed by calculating C-statistics and generating ROC curves with other measures of diagnostic tests.

**Results:**

All the models showed high negative prediction values (NPVs, 84 % to 92 %) and these did not differ between models or gender. The sensitivity of the models substantially changed based on the risk prediction thresholds (between 5–30 %); however, the NPVs and PPVs were relatively stable at various threshold levels. Hypertension and dyslipidaemia were significantly associated with CHD outcome in males and ABSI (a body shape index) in females. All models showed similar C-statistics (0.611–0.685, in both genders). Overall, the non-laboratory-based model showed better performance (0.685) in women but equal performance in men.

**Conclusions:**

Existing CVD risk prediction tools may identify future CHD cases with fairly good confidence on a short-term basis. The non-laboratory-based tool, using ABSI as a predictor, may provide better predictive accuracy among women.

## Background

Prediction of risk can greatly help in the management and prevention of cardiovascular diseases (CVDs) as well as in designing long-term policies and programs in this sector. It is now well-acknowledged that absolute risk assessment, based on the combined effect of multiple risk factors, yields better accuracy compared to the individual risk factor based approach in predicting CVD events [1, 2]. Absolute risk factor profiling was originally proposed in the landmark Framingham study [3, 4] and most of the later prediction tools [5–7] are adapted from the original one. Another important development in this area is the WHO/ISH 10-years CVD risk assessment chart proposed in 2007 [8] which was designed as a tool suitable for application in low resource settings.

Framingham scoring and its adaptations have been validated through various prospective and longitudinal studies [9–11], but those have been done almost solely in the context of developed societies. In contrast, a number of studies have been conducted with the WHO/ISH tool in developing countries [12–14], but those are almost exclusively cross-sectional studies and validations by prospective and longitudinal studies are lacking.

In recent years we have initiated a cohort in a peripherally located rural Bangladeshi population from which baseline data on individual and absolute CVD risk have been reported previously [15]. In the present communication, two laboratory and two non-laboratory-based models of absolute CVD prediction tools (based on adaptation of Framingham risk score, ‘with’ or ‘without cholesterol’ version of WHO/ISH tool, and a tool with the same risk factors as Framingham but with laboratory variables replaced by the best anthropometric predictive risk factor for CHD from this study, have been tested for a ‘proof of the concept’ on a short-term (2.5 years) basis. The outcome variable in this study is electrocardiographic evidence of coronary heart disease (CHD) which has been considered as a surrogate marker of CVDs in general [16–18]. The advantage of using ECG as a tool is its objectivity to avoid recall bias in this underdeveloped rural population with poor socioeconomic, educational and disease awareness status. Although 2.5 years is a limited period for risk predictivity, to the best of our knowledge, no study has yet been done with any tool on such a short-term basis and thus, the findings may be of interest for practicing clinicians.

## Methods

The original cohort was initiated in 2008 under the ‘BADAS-ORBIS Eye Care Project’. The cohort had 66,701 participants aged between 31–74 years in 2008. In 2011-12, a screening program was conducted using a questionnaire based tool developed as a part of the ‘WHO CVD risk management package for low- and medium – resource settings’ and following the recommendations of WHO [19]. From the remaining ‘screened negative’ participants (*n* = 62,538), a sub-cohort were recruited randomly. Initially 1000 participants were approached; out of them 563 (56.3 %) agreed to take part and provided data. The detailed description of the program is available elsewhere [15]. Following the case-cohort design with maximum 2.5 years of follow-up, from July 2012 to December 2014, another screening program was conducted using similar steps as in September 2011 to March 2012. CHD-related abnormalities were evidenced by ECG. In 2014, of the 63,708 eligible residents, 52,989 gave consent (participation rate 85.02 %) and 42 were ECG positive. In the sub-cohort 77.97 % (439/563) agreed, 18 were ECG positive and 27 did not complete all the biochemical tests of the study.

All the ECG positive and consented sub-cohort participants, using a structured, pretested, interviewer administrated questionnaire, were interviewed to obtain information on (i) socio-demographic characteristics, (ii) three days dietary intake history including fruit and vegetable intake [consumption assessed by a question that inquired the number of serving (medium portions) of any fruit or vegetable per day], (iii) smoking status including type of smoking and/or smokeless tobacco use, past smoking history; (iv) physical examination including blood pressure measurements with an oscillometric device after at least 5 min of rest and blood biochemistry. Height and weight were measured; body mass index (BMI) (kg/m^2^), waist circumference (WC), hip circumference (HC) and waist-hip-ratio (WHR) were calculated. ABSI was calculated as WC divided by BMI in power of 2/3 multiplied by height in power of 1/2 (WC/(BMI^2/3^ × height^1/2^)) [20].

Hypertension was categorized according to blood pressure (BP) readings by JNC-V definitions [21]: optimal (systolic, <120 mm Hg and diastolic, <80 mm Hg), normal blood pressure (systolic <120 to 129 mm Hg or diastolic <80 to 84 mm Hg), high normal blood pressure (systolic 130 to 139 mm Hg or diastolic 85 to 89 mm Hg), hypertension stage I (systolic 140 to 159 mm Hg or diastolic 90 to 99 mm Hg), and hypertension stage II–IV (systolic ≥160 or diastolic ≥100 mm Hg). When systolic and diastolic pressures fell into different categories, the higher category was selected for the purpose of classification. Blood pressure categorization was made dis regarding the use of anti-hypertension medication. Diabetes mellitus (DM) was considered as fasting blood glucose (FBG) ≥7.0 mmol/L and/or 2 h after 75-g oral glucose solution ≥11.1 mmol/L and pre-DM followed by the WHO guideline [22]. In addition, DM was defined by the use of insulin or oral anti-diabetic medication(s). Blood was drawn at the baseline examination after an overnight fasting, and ethylene diamine tetraacetic acid (EDTA) plasma was used for all cholesterol, triglyceride and HDL (mg/dl) measurements. All of them were determined according to the enzymatic colorimetric method, and LDL was estimated by Friedewald’s formula. Study subjects were followed up over a 2.5-years period for the development of CHD (includes angina pectoris, recognized and unrecognized myocardial infarction, coronary insufficiency, and coronary heart disease death). We collected binary information on smoking (Smoker/non-smoker). Current regular smoking was defined as at least one cigarette per day or smoked regularly during the previous 12 months.

We compared four risk prediction models: model 1: the Framingham laboratory-based model; model 2: ‘With’ cholesterol versions and model 3: ‘Without’ cholesterol version of the World Health Organization/International Society of Hypertension chart developed for estimating CVD risk for the South-East Asian Region D, and model 4: Non-laboratory-based model. We also checked how well these models could predict various levels of risks for cardiovascular events in the North Bengal Non-Communicable Disease Program (NB-NCDP) cohort. In model 1 we used the same risk factors as in the Framingham model: sex, age (years), systolic blood pressure (SBP; mm Hg), smoking status (past or current *vs* never), total cholesterol (TC), High-density lipoprotein (HDL), measured or reported diabetes status (yes/no), and current treatment for raised blood pressure (yes/no). In model 2, we used the same variables as the laboratory-based model (model 1) except HDL (same as WHO/ISH with cholesterol risk) and in model 3 we excluded TC and HDL (same as WHO/ISH without cholesterol risk). In model 4 we used the same risk factors as in model 1 but replaced TC and HDL with ABSI as an anthropometric indicator. This could be a unique model for NB-NCDP as we replaced anthropometric indicator based on maximum strength of association with CHD from our data set.

### Ethical consideration

The present study was carried out according to the guidelines laid down in the Declaration of Helsinki on medical ethics. All participants provided verbal consent in presence of witness [23] and the NB-NCDP study was approved by the Human Research Ethics Committee (HREC) of the University of New South Wales (HREC ref: ≠HC12621), Sydney, Australia and the Ethics Review Committee of the Diabetic Association of Bangladesh (BADAS).

### Ascertainment of cases (Outcome assessment)

To identify cases, history of chest pain indicating cardiovascular problems (diagnosed by a set of questions, approved by WHO CVD-risk management package for low- and medium – resource settings’ for CVD screening) [19], were collected and ECG was performed in suspected cases. To be identified as an MI case for overall CHDs, the participants need to fulfil two criteria, a) symptoms of cardiac ischaemia and b) development of unequivocal pathological Q wave in the ECG [24]. Persons already diagnosed with MI by physician during the follow–up period were also considered as cases.

### Statistical analysis of case-cohort data

Descriptive statistics of demographic and other variables were reported separately for cases and non-cases in the study as well as by gender. Independent samples *t*-test and chi-squared test were conducted for continuous and categorical variables respectively for between group comparisons.

The end point in this study was defined as myocardial infarction (MI) evidenced from ECG abnormality. To estimate risk we fitted the Cox proportional hazards model to the calculation hazard ratio for developing CHD (i.e., MI).

Before fitting Cox models we appropriately created the analytical dataset from case-cohort design. For each subject in the case-cohort study, follow-up time was split into two parts, the time before the exit time and the exit time. Each non-failure from the sub-cohort contributes one line of data to the analytic data set as censored observations. Failures from the main cohort contribute no information prior to their failure times. Thus, they contribute one line of data to the analytic data set as failures but only at their failure times. This is because of the assumption that failures outside the sub-cohort occur just after entering the subject into the study [25]. Failures from the sub-cohort contribute two lines to the analytic data set: as a censored observation prior to their failure times and as a failure at their failure time. To create a time “just before the exit time,” an amount (0.0001) less than the precision of exit times given in the data was subtracted from the actual failure time [26]. The robust standard error was estimated using “COVSANDWICH (aggregate)” option in SAS. From the fitted model we predicted absolute failure risk for each observation in our dataset. From the predicted risk we calculated the C-statistic and generated receiver operator characteristic (ROC) curves for each of the four models separately by gender. The C-statistic was calculated and compared across different models using the *roccomp* command in STATA version 13. Smoothed ROC curves were generated using PROC SGPLOT in SAS to distinguish the curves for different models. All the regression analyses were conducted separately for males and females.

We used SAS version 9.4 for fitting the Cox models as described in Langholza & Jiaob (2007) [26].

The predictive power of those four models was compared using C-statistic and ROC curves. We also calculated sensitivity, specificity, positive predictive value (PPV), negative predictive value (NPV) and the percentage correctly classified for this purpose. These parameters were calculated by using four different cut-off values (5 %, 10 %, 20 % and 30 %) of the predicted absolute risk.

We also calculated the “Net Benefit Fraction (NBF)”, defined as (TP − w × FP)/N, where TP is the number of true-positive decisions, FP is the number of false-positive decisions, N is the total number of the population, and w is a weight equal to the odds of the threshold (P treatment/(1 − P treatment)). This is considered as the harm/benefit ratio of treatment; for example, at the threshold of 10 %, the FP is valued at one-ninth of the TP [27]. Because the maximum net benefit equals the incidence rate of disease [28], given that all events are TP with no FP, we divided net benefit by the incidence rate. In this way, we defined the net benefit fraction as a simple relative utility index [29], which is the fraction of the incidence rate that could be predicted and prevented, appropriately regarding the usefulness of treatment for true positives and a negative weight for harmfulness of treatment in false positives.

Statistical analyses were performed by using SPSS for Windows, version 22 (SPSS, Inc., Chicago, Illinois), Stata, version 13 (StataCorp LP, College Station, Texas) statistical software and SAS. Two-sided *P* < 0.05 was considered statistically significant.

## Results

**Fig. 1:**
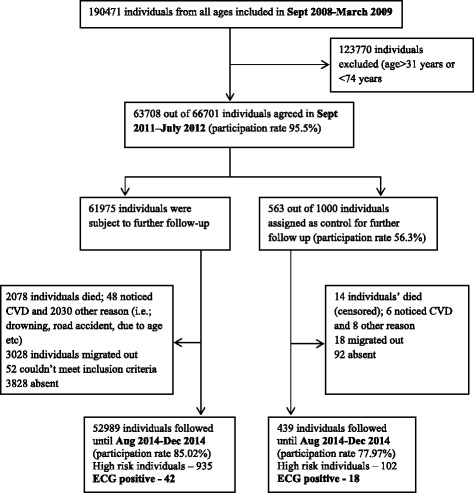
Case cohort follow up profile

The follow-up time ranged from 24–29 months (2—2.5 years, based on starting date of the follow-up of the sub-cohort and the date of ECG assessment) with a minimum of 2 years. The median follow-up time was 814 days with a range of 770 to 851 days. There were 60 incident cases of MI during this study follow-up period of 2.5 years. These 60 cases were generated from a total follow-up of 156337.5 person years (including 6 non-CVD related deaths) which translates in to an incidence rate of 38.38 cases per 100,000 person years. The overall characteristics of the population are listed in Table 1. By design, the NB-NCDP cohort was representative of the adult rural population in Bangladesh. Most participants were in middle age, mean age ± SD was 53.73 ± 10.71 years. The majority had no or only primary school education, poor vegetable and fruits intake, one third of participants were under weight and the majority had abnormal HDL. Overall, with the exception of a higher number of female cases (*p* < 0.016), higher rates of elevated DBP (*p* < 0.017), pre-HTN and HTN (*p* < 0.043) for cases, the CVD risk distribution was similar between controls and cases. The 54 deaths due to cardiovascular disease represented 2.58 % of all deaths in the cohort (Fig. 1).

**Table 1:** Distribution of socio demographic, behavioral, anthropometric, clinical and biochemical characteristics of the study participants

Variables^a^		Disease free^b^ (*n* = 394)	Total cases^b^ (*n* = 60)	*P* value^c^
Gender				
	Male	258 (65.5)	29 (48.3)	0.010
	Female	136 (34.6)	31 (51.7)	
Age (years) (M ± SD)		53.73 ± 10.71	53.90 ± 10.75	0.909
	31–45	115 (29.2)	19 (31.7)	0.927
	46–60	180 (45.7)	25 (41.7)	
	61 yrs & above	99 (25.1)	16 (26.7)	
Education				
	None or Primary	296 (75.1)	52 (86.7)	<0.001
	Secondary level and above	98 (24.9)	8 (13.3)	
Gross National Income (per capita, US$)				
	Low income (≤905)	204 (51.8)	30 (50.0)	0.858
	Lower-middle income (906–3595)	190 (48.2)	30 (50.0)	
Employment status				
	Unemployed/sacked from the present job/Retired/House maker/farmer	236 (59.9)	39 (65.0)	0.448
	Office work/Business/Skilled labour/Rickshaw puller/day labour/Others	158 (40.1)	21 (35.0)	
Behavioral risk factors				
Smoking Pattern				
	Non-smoker	276 (70.1)	45 (75.0)	0.543
	Smoker	118 (29.9)	15 (25.0)	
Smokeless tobacco				
	Non smokeless tobacco	242 (61.4)	46 (76.7)	0.022
	Regular smokeless tobacco	152 (38.6)	14 (23.3)	
Fruits intake pattern				
	Less than 1 servings/day	393 (99.7)	60.0 (100)	0.594
	1–2 servings/day	1 (0.3)	-	
Vegetables intake pattern				
	Less than 2 servings/day	205 (52.0)	39 (65.0)	0.071
	3–5 servings/day	189 (48.0)	21 (35.0)	
Anthropometric risk factors				
BMI (M ± SD)		20.0 ± 3.6	19.6 ± 3.3	0.298
	Underweight (BMI < 18.5)	139 (35.3)	25 (41.7)	0.256
	Normal (18.51–23.0)	176 (44.7)	26 (43.3)	
	Overweight and obese (>23.0)	79 (20.1)	9 (15.0)	
Waist circumference		80.46 ± 10.0	81.5 ± 10.9	0.489
	Normal (<0.90 male, <0.80 female)	300 (76.1)	37 (61.7)	0.021
	High risk (>0.90 male, >0.80 female)	94 (23.9)	23 (38.3)	
Waist Hip Ratio		0.93 ± 0.06	0.92 ± 0.06	0.916
	Normal (<0.95 male, <0.80 female)	185 (47.1)	22 (36.7)	0.065
	Moderate (0.96–1.0 male, 0.81–0.85 female)	57 (14.5)	7 (11.7)	
	High risk (>1.0 male, >0.85 female)	151 (38.4)	31 (51.7)	
Waist Height Ratio		0.51 ± 0.06	0.52 ± 0.07	0.404
	<=0.5 (non central fat distribution - pears)	195 (49.5)	26 (43.3)	0.375
	>0.5 (central fat distribution - apples)	199 (50.5)	34 (56.7)	
ABSI (m^11/6^/kg^2/3^) (M ± SD)		0.0868 ± 0.0065	0.0895 ± 0.0070	0.003
Clinical and biochemical risk factors				
Systolic blood pressure (mmHg)		115 ± 31	119 ± 24	0.273
	Normal (≤140 mmHg)	375 (95.2)	57 (95.0)	0.394
	High (≥140 mmHg)	19 (4.8)	3 (5.0)	
Diastolic blood pressure (mmHg)		73 ± 14	77 ± 13	0.55
	Normal (≤90 mmHg)	384 (97.5)	52 (86.7)	0.017
	High (≥90 mmHg)	10 (2.5)	8 (13.3)	
Hypertension				
	Normotensive	340 (86.3)	47 (78.3)	0.043
	Pre-hypertensive	49 (12.4)	10 (16.7)	
	Hypertensive	5 (1.3)	3 (5.0)	
Biochemical risk factors				
Fasting blood glucose (mmol/l)		4.44 ± 1.25	4.53 ± 1.02	0.555
2 hrs after 75gm glucose (mmol/l)		6.76 ± 2.09	6.83 ± 2.66	0.855
Glycemic Status				
	Non diabetic	280 (81.6)	43 (76.8)	0.368
	Pre-diabetic	55 (16.0)	11 (19.6)	
	Diabetic	8 (2.3)	2 (3.6)	
Cholesterol (mg/dl)		179 ± 44	179 ± 53	0.975
	<200 normal	265 (75.9)	48 (85.7)	0.427
	200.01–240 border line high	56 (16.0)	2 (3.6)	
	>240.01 high	28 (8.0)	6 (10.7)	
Triglyceride (mg/dl)		145 ± 100	145 ± 67	0.991
	<150 normal	242 (69.2)	39 (69.6)	0.787
	150.01–200 border line high	61 (17.5)	11 (19.6)	
	>200.01 high	46 (13.2)	6 (10.7)	
HDL (mg/dl)		38 ± 10	37 ± 8	0.219
	Normal (male >40, Female >50)	14 (4.0)	1 (1.8)	0.705
	Risk (male < 40, Female < 50)	335 (96.0)	55 (98.2)	
LDL (mg/dl)		114 ± 31	114 ± 44	0.997
	Normal (LDL < 100)	135 (38.8)	23 (41.1)	0.879
	Near normal (LDL ≥ 100.01 & < 130)	123 (35.3)	21 (37.5)	
	High (LDL ≥ 130.01 & < 190)	82 (23.6)	8 (14.3)	
	Very high (LDL > 190.01)	8 (2.3)	4 (7.1)	

^a^Values expressed as numbers and percentages in parentheses or mean ± SD, as appropriate; SD, standard deviation; yrs, years; ^b^All the disease free participants came only from the sub-cohorts but the cases came from both the main and sub-cohorts; ^c^For continuous variables *p*-values were obtained by doing independent samples *t*-test and for categorical variable from chi-squared test; Significance between normal and total cases

Table 2 shows summary statistics for risk factors used in risk models. Female participants were, on average, five years younger than males and had a higher rate of abnormal total cholesterol. On contrary, males had higher smoking and BP treatment rates. The remaining risk factors were similar between the sexes.

**Table 2:** Summary statistics for risk factors used in risk models (All cases identified from main cohort and all participants from sub-cohort)

Characteristics	^a^Women (*n* = 167)	^b^Men (*n* = 287)
Age, mean (SD), y	50.8 (10.4)	55.5 (10.5)
Total-C, mean (SD), mg/dl	178.9 (56.2)	178.9 (39.3)
HDL-C, mean (SD), mg/dl	37.2 (8.8)	38.0 (10.1)
Systolic BP, mean (SD), mm Hg	113.4 (23.2)	115.0 (20.9)
BP treatment, n (%)	14 (8.4)	27 (9.4)
Smoking, n (%)	23 (13.8)	110 (38.3)
Diabetes, n (%)	21 (12.6)	57 (19.9)
ABSI (m^11/6^/kg^2/3^), mean (SD)	0.0893 (0.0069)	0.859 (0062)

^a^among women, 22 cases from main cohort and 9 cases from the sub-cohort

^b^among men, 20 cases from main cohort and 9 cases from the sub-cohort

Table 3 shows hazard ratio with 95 % confidence interval and *p* values from the Cox regression models predicting cardiovascular disease events by sex. All four predictive models (i.e., model 1, model 2, model 3 and model 4) showed almost similar pattern with risk distribution. It showed that systolic blood pressure and dyslipidaemia (i.e., TC and HDL) for men in all four models and ABSI for women in model 4 was significant. In women, model 4 showed fair predictive power, with a c statistic (95 % CI) of 0.685 (0.581–0.789). The C-statistic of model 1, 2 and 3 were 0.634 (0.526–0.741); 0.626 (0.521–0.731) and 0.611 (0.506–0.717) respectively. The four c statistics were not significantly different (*χ*^2^ = 1.08, with 3 DF *P* = 0.7814). In men, the C-statistic for model 1 was 0.675 (0.575–0.775) and for model 2, 3 and 4 were 0.644 (0.541–0.747), 0.631 (0.528–0.734) and 0.627 (0.525–0.728) respectively. They were also not significantly different (*χ*^2^ = 0.54, with 3 DF *P* = 0.9092).

**Table 3:** Hazard ratios of CHD (only MI) from multivariable Cox proportional hazards model

Variable		Women			Men		
		HR	95 % CI	*P*	HR	95 % CI	*P*
Model 1 (Laboratory based)^a^							
	Age (in 10 yrs)	0.99	0.71–1.38	0.968	1.38	0.97–1.98	0.078
	Total cholesterol (mg/dl in 10 yrs)	0.99	0.88–1.12	0.930	1.13	1.03–1.26	0.015
	HDL cholesterol (mg/dl in 10 yrs)	0.77	0.39–1.53	0.455	0.50	0.26–0.98	0.042
	Systolic blood pressure (mm Hg in 10 yrs)	1.18	0.96–1.55	1.452	1.52	1.23–1.89	0.0001
	History of blood pressure treatment	0.78	0.15–3.99	0.763	1.54	0.45–5.30	0.490
	Current smoker	0.77	0.27–2.23	0.628	1.88	0.93–3.81	0.081
	Diabetes	1.22	0.46–3.26	0.694	1.34	0.61–2.9	0.466
Model 2 (WHO with cholesterol)^b^							
	Age (in 10 yrs)	1.10	0.70–1.73	0.683	1.39	0.87–2.21	0.168
	Total cholesterol (mg/dl in 10 yrs)	0.97	0.87–1.08	0.614	1.01	0.89–1.15	0.878
	Systolic blood pressure (mm Hg in 10 yrs)	1.21	0.91–1.61	0.187	1.62	1.22–2.15	0.0008
	History of blood pressure treatment	0.80	0.16–3.92	0.781	1.80	0.51–6.31	0.357
	Current smoker	0.53	0.11–2.64	0.437	1.54	0.61–3.93	0.363
	Diabetes	1.58	0.47–5.23	0.455	1.37	0.51–3.70	0.534
Model 3 (WHO without cholesterol)^c^							
	Age (in 10 yrs)	1.05	0.67–1.63	0.833	1.45	0.90–2.34	0.123
	Systolic blood pressure (mm Hg in 10 yrs)	1.11	0.76–1.62	0.606	1.77	1.35–2.32	<0.0001
	History of blood pressure treatment	0.44	0.06–3.10	0.412	1.94	0.48–7.82	0.348
	Current smoker	0.52	0.11–2.56	0.421	1.56	0.61–3.98	0.353
	Diabetes	1.44	0.43–4.83	0.556	1.41	0.53–3.78	0.488
Model 4 (Non-laboratory based)^d^							
	Age (in 10 yrs)	0.83	0.51–1.34	0.448	1.36	0.89–2.08	0.149
	Systolic blood pressure (mm Hg in 10 yrs)	1.13	0.86–1.48	0.392	1.57	1.21–2.04	0.0007
	History of blood pressure treatment	1.65	0.36–7.51	0.516	1.79	0.51–6.28	0.369
	Current smoker	0.84	0.21–3.41	0.827	1.74	0.78–3.87	0.175
	Diabetes	0.88	0.27–2.84	0.827	1.16	0.46–2.90	0.754
	ABSI^e^ (from 1 SD)	3.20	1.60–6.42	0.001	1.09	0.53–2.22	0.817

*HR* hazard ration, *yrs* years, *SD* standard deviation

^a^C statistics (95 % CI): 0.634 (0.527–0.710) for women; 0.675 (0.575–0.775) for men

^b^C statistics (95 % CI): 0.626 (0.521–0.731) for women; 0.644 (0.541–0.746) for men

^c^C statistics (95 % CI): 0.611 (0.506–0.717) for women; 0.631 (0.528–0.734) for men

^d^C statistics (95 % CI): 0.685 (0.581–0.789) for women; 0.627 (0.525–0.728) for men

^e^ABSI (1 ± SD, male 0.0062 and female 0.0069)

**Fig 2:**
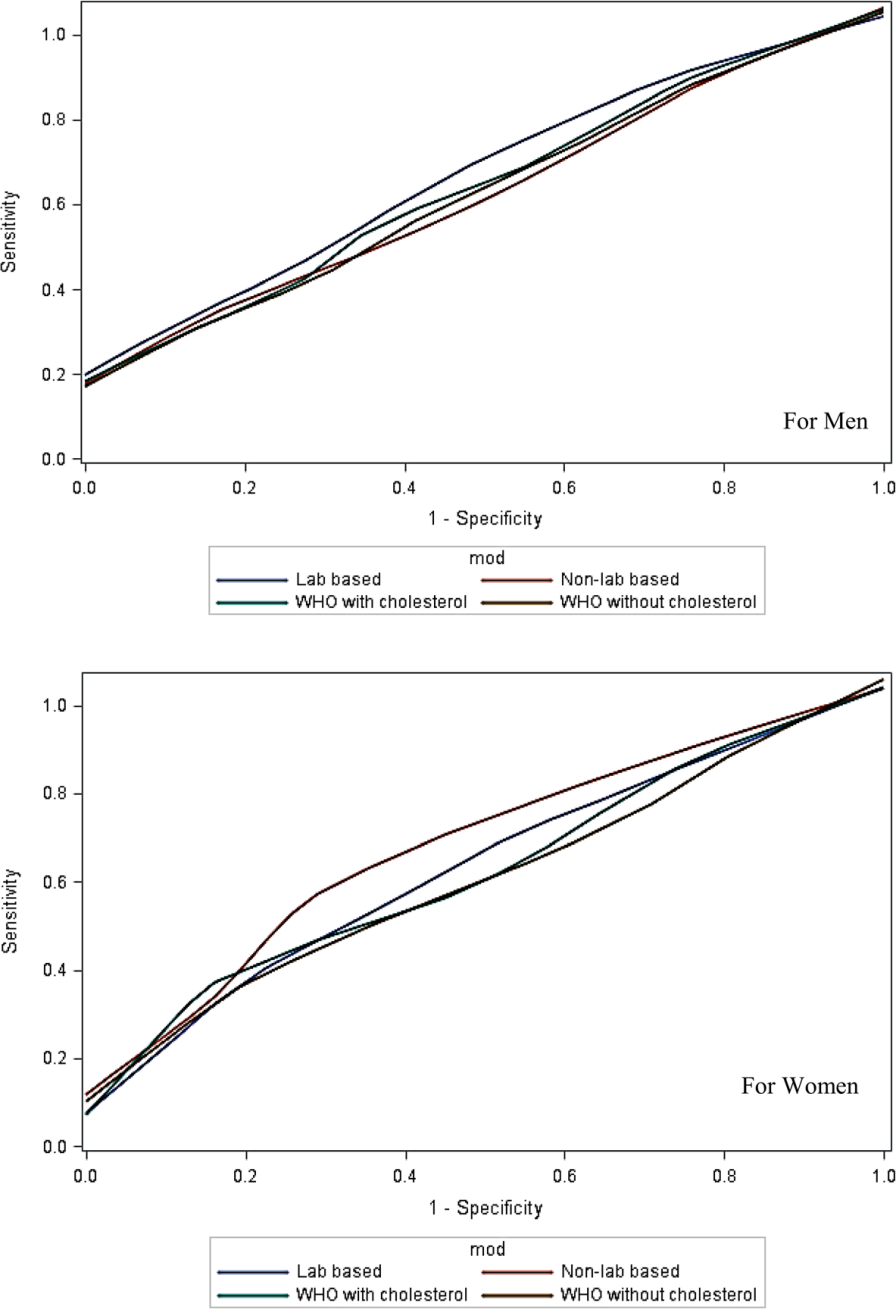
ROC curves for men (top) and women (bottom) for model 1 (laboratory-based), model 2 (WHO/ISH with cholesterol) and model 3 (WHO/ISH without cholesterol) and model 4 (non-laboratory-based) methods for prediction of cardiovascular disease (based on maximum 2.5 months observation period)

The ROC curves show a large amount of overlap in the predictive discrimination of the four models for both women and men. Adding ABSI to the non-laboratory-based model instead of total cholesterol did not improve the predictive discrimination in either sex (Fig. 2).

An ECG-based definition of cardiovascular disease, that included only MI cases, was used, but the difference between the four models remained small with narrower endpoints. The analysis with cardiovascular deaths only, where the possibility of misclassification is kept to a minimum, resulted in C-statistics of 0.675, 0.644, 0.631 and 0.627 for model 1–4 respectively in the men, with similar results for women. These C-statistics were not significantly different.

The predictive discrimination of all four models against the various screening test characteristics is shown in Table 4. There was no significant difference in any of the characteristics between the four models at each of the risk thresholds tested for women or men. The sensitivity and specificity of both tests were also similar for each model at each risk threshold. Sensitivity was in between 65–69 % (men) and 84–90 % (women) at the lowest threshold (5 %, 2.5-year risk) and less than 29 % for women and 21 % for men at the highest threshold (30 %, 2.5-year risk). Considering all four models, among men, only 11 % developed CVD events during follow-up (positive predictive value, PPV), whereas, of those categorised at low-risk level, 92 % remained event free during the follow-up (negative predictive value, NPV). On the other hand, among women PPV was 20 % and NPV was 90 %. When the threshold was greater than 30 %, the positive predictive value for all models was roughly 18 % and 11 % and the negative predictive value greater than 81 % and 90 % for women and men respectively. The results for the alternative analysis using the threshold of 10 % and 20 % are shown in Table 4.

**Table 4:** Predictive discrimination of four models at different cut off values of cardiovascular risk thresholds over 2.5-year of follow-up

Cut-off values		5 %	10 %	20 %	30 %
Women					
Sensitivity (95 % CI)					
	Model 1	87.1 (70.2–96.4)	71.0 (52.0–85.8)	54.8 (36.0–72.7)	41.9 (24.5–60.9)
	Model 2	90.3 (74.2–98.0)	67.7 (48.6–83.3)	48.4 (30.2–66.9)	35.5 (19.2–54.6)
	Model 3	87.1 (70.2–96.4)	71.0 (52.0–85.8)	45.2 (27.3–64.0)	29.0 (14.2–48.0)
	Model 4	83.9 (66.3–94.5)	77.4 (58.9–90.4)	54.8 (36.0–72.7)	38.7 (21.8–57.8)
Specificity (95 % CI)					
	Model 1	24.7 (17.9–32.5)	40.4 (32.4–48.8)	57.5 (49.1–65.7)	69.9 (61.7–77.2)
	Model 2	24.8 (17.3–31.7)	42.5 (34.3–50.9)	53.4 (45.0–61.7)	67.1 (58.9–74.7)
	Model 3	28.1 (21.0–36.1)	38.4 (30.4–46.8)	53.4 (45.0–61.7)	65.8 (57.5–73.4)
	Model 4	29.5 (22.2–37.6)	43.2 (35.0–51.6)	61.0 (52.5–68.9)	70.5 (62.5–77.8)
Positive predictive value (95 % CI)					
	Model 1	19.7 (13.4–27.4)	20.2 (13.1–28.9)	21.5 (13.1–32.2)	22.8 (12.7–35.8)
	Model 2	20.1 (13.8–27.8)	20.2 (12.8–28.9)	18.1 (10.5–28.0)	18.6 (9.7–30.9)
	Model 3	20.5 (13.9–28.3)	19.6 (12.7–28.2)	17.1 (9.7–27.0)	15.3 (7.2–27.0)
	Model 4	20.2 (13.6–28.1)	22.4 (14.9–31.5)	23.0 (14.0–34.2)	21.8 (11.8–35.0)
Negative predictive value (95 % CI)					
	Model 1	90.0 (76.3–97.2)	86.8 (76.4–93.8)	85.7 (77.2–92.0)	85.0 (77.3–90.9)
	Model 2	92.1 (78.6–98.3)	86.1 (75.9–93.1)	83.0 (73.8–89.9)	83.1 (75.0–89.3)
	Model 3	91.1 (78.8–97.5)	86.2 (75.3–93.5)	82.1 (72.9–89.2)	81.4 (73.1–87.9)
	Model 4	89.6 (77.3–96.5)	90.0 (80.5–95.9)	86.4 (78.2–92.4)	84.4 (76.8–90.4)
Men					
Sensitivity (95 % CI)					
	Model 1	65.5 (45.7–82.1)	51.7 (32.5–70.6)	34.5 (17.9–54.3)	24.1 (10.3–43.5)
	Model 2	69.0 (49.2–84.7)	51.7 (32.5–70.6)	31.0 (15.3–50.8)	20.7 (8.0–39.7)
	Model 3	69.0 (49.2–84.7)	58.6 (38.9–76.5)	24.1 (10.3–43.5)	20.7 (8.0–39.7)
	Model 4	72.4 (32.1–43.9)	51.7 (32.5–70.6)	24.1 (10.3–43.5)	24.1 (10.3–43.5)
Specificity (95 % CI)					
	Model 1	44.1 (45.7–82.1)	58.8 (52.7–64.7)	73.2 (67.5–78.3)	79.8 (74.5–84.4)
	Model 2	41.5 (35.6–47.6)	54.0 (47.9–60.1)	72.1 (66.3–77.3)	82.4 (77.3–86.7)
	Model 3	38.6 (32.8–44.7)	53.7 (47.6–59.7)	73.2 (67.5–78.3)	83.1 (78.1–87.3)
	Model 4	37.9 (32.1–43.9)	52.9 (46.8–59.0)	72.1 (66.3–77.3)	81.3 (76.1–85.7)
Positive predictive value (95 % CI)					
	Model 1	11.1 (6.8–16.8)	11.8 (6.8–18.7)	12.0 (5.9–21.0)	11.3 (4.7–21.9)
	Model 2	11.2 (7.0–16.7)	10.7 (6.1–17.1)	10.6 (5.0–19.2)	11.1 (4.2–22.6)
	Model 3	10.7 (6.7–16.0)	11.9 (7.1–18.4)	8.8 (3.6–17.2)	11.5 (4.4–23.4)
	Model 4	11.1 (7.0–16.4)	9.9 (5.5–16.0)	8.4 (3.5–16.6)	12.1 (5.0–23.3)
Negative predictive value (95 % CI)					
	Model 1	92.3 (86.3–96.2)	92.0 (86.9–95.5)	91.3 (86.7–94.7)	90.8 (86.4–94.1)
	Model 2	92.6 (86.5–96.6)	91.3 (85.8–95.2)	90.7 (86.1–94.3)	90.7 (86.4–94.0)
	Model 3	92.1 (85.5–96.3)	92.4 (87.1–96.0)	90.0 (85.3–93.7)	90.8 (86.5–94.1)
	Model 4	92.8 (86.3–96.8)	90.6 (84.9–94.6)	89.9 (85.1–93.6)	90.9 (86.6–94.2)

*Abbreviations*: *CI* confidence interval; Model 1, Laboratory based model same as Framingham Risk Score; Model 2, Non laboratory based model; Model 3, WHO/ISH with Cholesterol; Model 4, WHO/ISH without Cholesterol

From a clinical performance view point, all models have nearly the same net benefit fraction [14 % for women and 5.0 % for men] at lowest threshold (5 %). The net benefit fractions were 13.9 %, 14.5 %, 14.1 % and 13.5 %; 4.8 %, 5.1 %, 4.8 % and 5.2 % of the incidence of women and men, and model 1, 2 3 and 4 respectively at 5 % threshold level. For higher threshold, NBF values are truly low or negative. Due to the low predictive power of the models the curve showed a poor net benefit at the lowest threshold (<5 %) with respect to the different predictive models over the whole range of values. The curve performances were worse in men (results are not shown here).

## Discussion

The present data show that short-term (2.5 years) predictive discrimination values of the models do not differ significantly among them within either sex. All models have quite good NPVs but poor PPVs. The non-laboratory based models (e.g., model 3 and 4), that used easily obtainable information from any participant even from a single outpatient visit, can predict CVD outcomes with the same degree of accuracy as the laboratory-based tools that require HDL and/or total cholesterol and thus become expensive and difficult to be applied in some settings. From the overall analysis the newly proposed non-lab based model (which includes ABSI, a new anthropometric indicator) showed better performance in women.

These study findings indicate a quite high performance of all the four prediction tools in identifying subjects who will not develop CHD on a short-term (around 2.5 years) basis. The conclusion is based on the 84 % to 92 % NPVs with various models at different threshold levels. It should be noted that the sensitivity and specificity of the different tools vary considerably depending on the risk threshold chosen. Generally, the sensitivity is seen to decrease with increasing risk threshold while specificity behaves in the opposite manner. In contrast to sensitivity and specificity, the NPV varies little between the tools at any given risk threshold levels. There is still debate at which threshold level of CVD risk a clinical intervention should be made [30]. Some authors suggest a cut-off value of 20 % [3], but a cut-off value as low as 5 % has also been suggested [31]. A consistent NPV irrespective of the threshold levels will be helpful for the clinical decision making process. The ability of the present models in identifying the true negative (i.e. not to be treated) subjects could be useful to the clinicians in the context of the prevailing practices regarding CVDs. Based on individual risk factor analysis, over-treatment has been reported to be an equal problem to under-treatment among persons with CVD risk factors [32]. In Bangladesh, although there is not yet any published study, from empirical experience and from personal communication with a few practicing cardiologists in Dhaka it seems that over-treatment is an equal (if not greater) problem compared to under-treatment due to unregulated clinical practices (even by unqualified practitioners) and aggressive marketing of drugs. Accordingly, a fairly accurate decision on non-intervention has a positive contribution on an individual as well as population levels.

On the contrary to NPV, the PPVs of the present tools are remarkably low and they vary between men (around 10 %) and women (around 20 %). Like NPV, the values are fairly stable at various risk threshold levels. The performances of the tools, thus, are poor in identifying the true positive cases (i.e., subjects who should have medical treatment to reduce the chance of progression to CHDs). Again, the PPVs do not vary among the four models (Table 4).

The best method for analysing and reporting the performance of risk prediction tools in order to guide clinical decision making is still a subject of debate in the literature. Various authors have proposed NBF [27, 30] and decision curve analysis [28, 30] as alternate procedures in this respect. Until the suitability of these suggestions is fully established, application of the traditional views regarding PPV and NPV (based on clinical and economic benefit/harm of an intervention) should be continued. A close look at the findings of the present study shows that the clinicians will have an additional benefit for around 10 % of male and 20 % of female cases regarding the initiation of treatment; in the remaining cases, they will need to decide on their own judgment based on individual risk factors. However, the current prediction models have good NPV values and therefore may assist clinical decision making on which individuals do not require risk factor treatment beyond lifestyle advices. A unique situation with CVD risk factors is that all subjects with CVD risk are strongly advised to pursue healthy nutritional habits and lifestyle. Accordingly, whatever decision is made by the clinicians based on PPV and/or NPV, all subjects are advised to pursue practices which potentially prevent CVDs. In addition to clinical settings, public health programs are increasingly promoting healthier nutrition and lifestyle to reduce the risk of CVDs and thus subjects not requiring clinical intervention based on absolute risk assessment should still be exposed to health promotion messages.

It is worthwhile to note that the predictive performance of the non-laboratory-based models (3 & 4) did not vary from those of the laboratory-based ones (model 1 & 2). For women, model 4 (the Framingham adapted new model in NB-NCDP) had a higher C-index (95 % CI) of 0.685 (0.581–0.789) compared to the other three models [0.634 (0.526–0.741); 0.626 (0.521–0.731) and 0.611 (0.506–0.717) for model 1, 2 and 3 respectively], although the differences are not statistically significant (*p* = 0.7814 in men and 0.9092 in women). Model 4 also had a higher NPV in women. It is interesting to note that, in the Cox model, hazard ratios showed that BP (*p* < 0.001) and lipid profile (*p* < 0.015) are consistently associated with CHD outcome in men, but in women the association is shifted to ABSI (<0.0001). Inclusion of ABSI in the model may be the underlying reason for the higher C-index as well as NPV obtained with this tool.

The strengths of the current study include its case-cohort design and use of appropriate analytical techniques (e.g., calculation of C-statistics from a Cox model) taking into consideration of the subtlety in the study design. Inclusion of detailed follow-up data and availability of major anthropometric and other traditional cardiovascular risk factors were additional strengths of this study. These facilitated the independent comparison of different anthropometric findings to identify the best measure associated with CHD. Although use of ECG has increased the objectivity in diagnosing CHD, a major limitation in this study is that only CHD has been used as a marker of CVDs. In one study 85 % of the CVDs reported were ascribed to CHDs [30]. Still exclusion of non-CHD CVDs may be one reason for which we have a very low incidence rate of CVD cases compared to other studies that included MI and other cardiovascular events. A comprehensive clinical assessment by clinicians was not done during data collection in this study which might detect some CVDs other than MIs. In the absence of any evidence from the present population, it is difficult to ascertain the degree of conformity of the present findings with the overall incidence of CVD events. It is quite likely that we have underestimated the true rate. It is also possible that we have underestimated the incidence of CHD as only those participants with clinical and ECG features of myocardial infarction were included as cases. Another limitation is that, like other studies [3, 33], we used total cholesterol and HDL, but the lab-based studies did not improve predictive performance of the models (i.e., model 1 & 2) over the non-lab-based ones (models 3 & 4). The C-statistics of both laboratory-based and non-laboratory-based models in prediction of CVD were <0.70, though there was some outcome misclassification which is independent of the explanatory variable that would give a non-differential error. That error would pull the association towards null, which in turn, would jeopardise the predictive power of the models. Moreover, the small number of cases in the cohort also could be a reason of non-significant association with the known risk factors. Our sample size calculation was based on the minimum requirement of 5 cases per explanatory variables in the predictive model. We had only 29 cases in males and 31 in females. Thus, the total sample size was minimum for Model 2, 3 and 4, and less than required for Model 1 which limits the ability to test the performance of clinical prediction of these four models in this setting. However, even if the laboratory-based model was marginally improved (by C-statistics, over non-lab model), it is still an open question whether the additional benefit would be justified in the context of resource limited developing settings considering the involvement of additional cost and logistics.

## Conclusion

In conclusion, ‘Not to be treated for CVD risk’ cases, may be identified with fairly good confidence by using the most commonly used CVD risk prediction tools based on short-term prediction. A newly proposed non-laboratory-based tool, using the overall obesity marker ABSI as a key variable, seems to be an alternate with equal performance in men and slightly better performance in women. It would be worthwhile to follow the cohort for exploring and comparing the predictive ability of these four models regarding CVD outcome in the longer term.

## Abbreviations

ABSI, A body shape index; BMI, Body mass index; BP, Blood pressure; CHDs, Coronary heart diseases; Cox PH, Cox proportional hazards; CVDs, Cardiovascular diseases; DM, Diabetes mellitus; ECG, electrocardiography; EDTA, Ethylene diamine tetraacetic acid; FBG, Fasting blood glucose; HC, Hip circumference; HDL, High-density lipoprotein; HREC, Human Research Ethics Committee; MI, Myocardial infarction; NBF, Net benefit fraction; NB-NCDP, North Bengal non-communicable disease program; NPVs, Negative prediction values; PPVs, positive prediction values; SBP, Systolic blood pressure; TC, Total cholesterol; WC, Waist circumference; WHR, Waist-hip-ratio.
